# Plan optimization with L0-norm and group sparsity constraints for a new rotational, intensity-modulated brachytherapy for cervical cancer

**DOI:** 10.1371/journal.pone.0236585

**Published:** 2020-07-28

**Authors:** Hojin Kim, Young Kyung Lim, Youngmoon Goh, Chiyoung Jeong, Ui-Jung Hwang, Sang Hyoun Choi, Byungchul Cho, Jungwon Kwak

**Affiliations:** 1 Department of Radiation Oncology, Asan Medical Center, Seoul, Korea; 2 Proton Therapy Center, National Cancer Center, Goyang, Korea; 3 Department of Radiation Oncology, Chungnam National University Hospital, Daejeon, Korea; 4 Department of Radiation Oncology, Korea Cancer Center Hospital, Seoul, Korea; 5 Department of Radiation Oncology, Asan Medical Center, University of Ulsan College of Medicine, Seoul, Korea; University Hospital Zurich, SWITZERLAND

## Abstract

The aim of this work is to build a framework that comprehends inverse planning procedure and plan optimization algorithm tailored to a novel directional beam intensity-modulated brachytherapy (IMBT) of cervical cancer using a rotatable, single-channel radiation shield. Inverse planning is required for finding optimal beam emitting direction, source dwell position and dwell time, which begin with creating a kernel matrix for each structure based on Monte-Carlo simulated dose distribution in the rotatable shield. For efficient beam delivery and less transit dose, the number of source dwell positions and angles needs to be minimized. It can be solved by L0-norm regularization for fewest possible dwell points, and by group sparsity constraint in L2,*p*-norm (0≤*p*<1) besides L0-norm for fewest active applicator rotating angles. The dose distributions from our proposed algorithms were compared to those of conventional tandem-based intracavitary brachytherapy (ICR) plans for six cervical cancer patients. The algorithmic performance was evaluated in delivery efficiency and plan quality relative to the unconstrained algorithm. The proposed framework yielded substantially enhanced plan quality over the conventional ICR plans. The L0-norm and (group sparsity+L0-norm) constrained algorithms reduced the number of source dwell points by 60 and 70% and saved 5 and 8 rotational angles on average (7 and 11 angles for highly modulated cases), relative to the unconstrained algorithm, respectively. Though both algorithms reduced the optimal source dwell positions and angles, the group sparsity constrained optimization with L0-norm was more effective than the L0-norm constraint only, mainly because of considering physical constraints of the new IMBT applicator. With much fewer dwell points compared to the unconstrained, the proposed algorithms led to statistically similar plan quality in dose volume histograms and iso-dose lines. It also demonstrated that the plan optimized by rotating the applicator resulted in much better plan quality than that of conventional applicator-based plans.

## Introduction

Brachytherapy has strong therapeutic advantages because it uses the radiation emitted from radioactive isotopes which are placed in or adjacent to a tumor. Many of the clinical protocols used to treat patients with cervical cancer combine high-dose-rate (HDR) intracavitary brachytherapy (ICR) [1–7] with external beam radiation therapy (EBRT). Recent advances in brachytherapy have been focused on three-dimensional (3D) computed tomography (CT) and magnetic resonance (MR) imaging [8–10] to enhance the therapeutic relevance. With the help of 3D imaging, we can better visualize the location and shape of tumor as well as surrounding organs at risk (OAR), but conformal and non-invasive treatment techniques are still lacking.

As the conventional tandem applicators for cervical cancer treatment have an isotropic dose distribution, the tumor control ability must be limited when the tumor volume is extended asymmetrically along the cervical canal. The interstitial needles or seed implants are known as being the conformal therapeutic methods for cervical cancer. Thus, the available option for the extended disease is to combine the intracavitary with the interstitial needles [11]. In fact, however, plan quality of the interstitial brachytherapy highly relies on the proficiency of the physician for the operation. Contrarily, the intracavitary brachytherapy has very little dependence on the empirical aspects.

In an effort to enhance the therapeutic applicability, the concept of intensity modulation was incorporated into intracavitary brachytherapy [12,13]. The intensity-modulated brachytherapy (IMBT) techniques using radiation shields have begun to emerge [14–19]. Shi *et al* [17] investigated the possibility of 3D IMBT for dose calculation modeling in treatment planning. However, they encountered such challenges in implementing IMBT as the need for a radiation source shield with thickness on the order of centimeters and prolonged treatment time. Another study by Lin *et al* [16] fabricated a radiation source in the form of a gold shield that yielded radiation with directional bias. It was demonstrated in [19] that IMBT for clinical applications by constructing a tandem applicator with directional modulation has high potential. The applicator was designed to load multiple channels with 60-degree angular spacing in order to create anisotropic, asymmetric dose distributions. These studies demonstrated the superiority of IMBT over the conventional tandem applicator for dose distribution, while the degree of freedom for the beam angular frequency could be limited.

Recently, our collaborative group developed a novel tandem applicator designed to generate anisotropic dose distribution [20]. The unique feature of the applicator enables the radiation source to deliver the dose in selected transverse directions by discretely rotating the radiation shield inside the tandem applicator, such that it modulates the beam weights with an angular precision less than 0.1^o^ mechanically. As the developed applicator is single-channeled, and the radiation source needs to travel back and forth frequently, the number of source dwell positions and beam angles might increase significantly. The higher number of source dwell points and applicator angles does not only degenerate the treatment delivery efficiency, but also raises the transit dose to the patients and dose uncertainty. Thus, to promote both delivery efficiency and patient safety in actual treatment, it is essential for the plan optimization algorithm to focus on the fewest possible source dwell points and rotational angles without a compromise in the plan quality. These aspects demand inverse planning with a new optimization strategy differentiated from the existing, which can be achieved by adopting sparse signal-reconstruction algorithms [21–27] and group sparsity algorithm [28, 29].

This work presents a new plan optimization framework customized for the novel tandem applicator, while applicable for the other IMBT techniques. It first focuses on how to build a structure-based system matrix applicable to inverse planning. Subsequently, new optimization algorithms tailored to the novel applicator are proposed with the aim of achieving: 1) the fewest possible optimal source dwell points by L0-norm regularization and 2) minimum rotation angles by group sparsity algorithms in L2,*p*-norm (0≤*p*<1) besides the L0-norm. The proposed framework was evaluated using six cervical cancer cases, relative to the conventional tandem-based intracavitary plan. The performance of the algorithms with appropriate constraints was compared against unconstrained algorithm in terms of the number of optimal source dwell points and the resultant plan quality.

## Materials and methods

### Creation of dose kernel matrix

**Fig 1:**
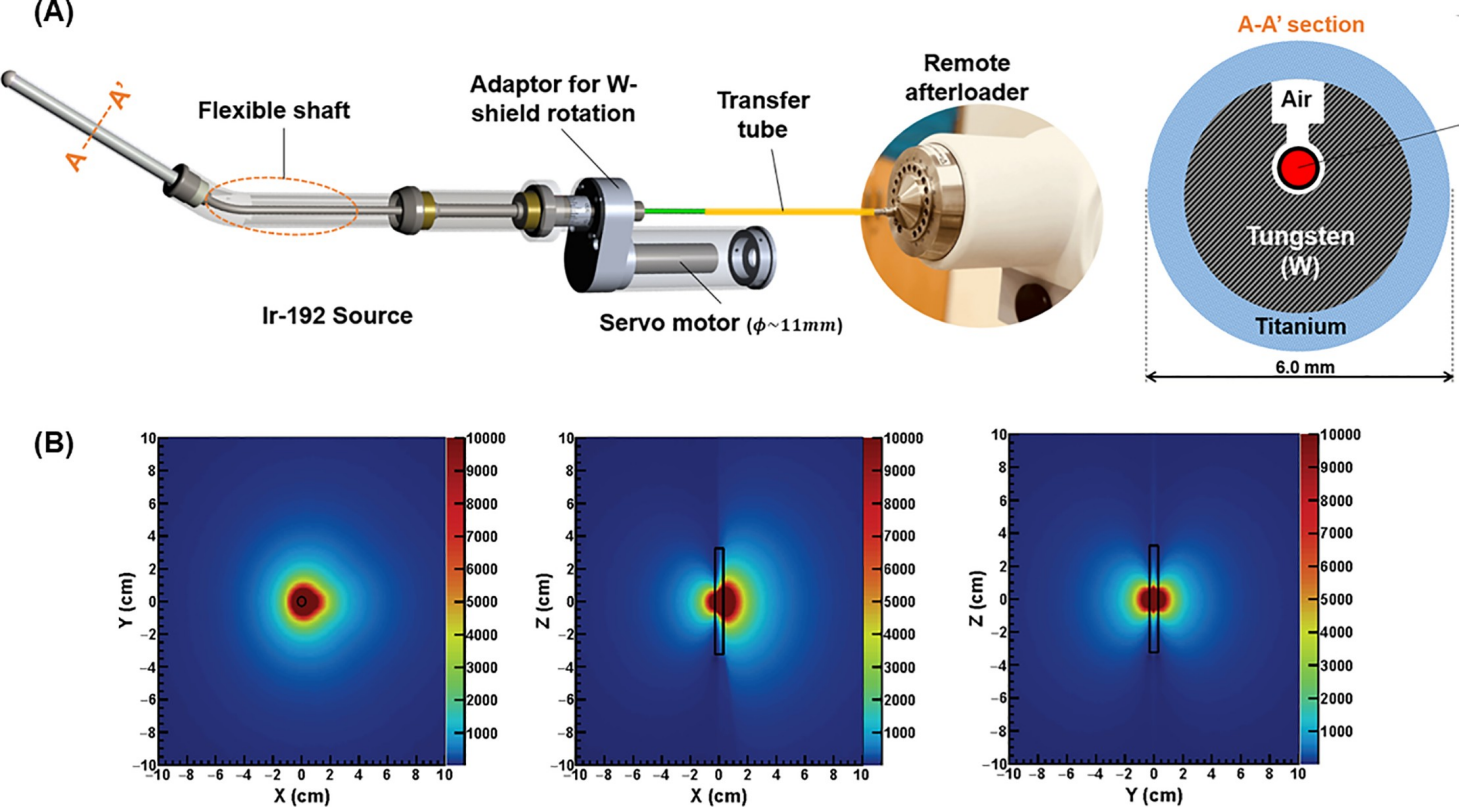
Novel rotatable tandem applicator and estimated dose distribution. (A) Novel, rotatable tandem applicator. (Bottom) Monte-Carlo-simulated dose distribution in water medium for ^192^Ir radiation source with the new tandem applicator, which is orthogonal to the axial plane, irradiated to the left-hand side in the image plane.

Fig 1(A) shows the novel rotational tandem applicator for IMBT. The applicator has a 7-cm long cylindrical tungsten shield with a grooved, rotatable tube driven by a servo motor with one end connected to a hollow flexible shaft. Assuming the radiation source is ^192^Ir, Monte-Carlo (MC) simulation can produce the dose distribution for a specific source position and rotation [30]. According to the AAPM TG-43 task group report [31, 32], dose calculation for brachytherapy planning can be conducted on the water medium. The heterogeneity possibly originated from the radiation shield and cavity in applicator was considered in kernel calculation for MC based simulation. Fig 1(B) depicts the dose distribution when the source is orthogonal to the axial plane, and irradiated to the left-hand side defined as 0^o^ in this work.

The MC-calculated dose distribution will be used for the inverse planning framework. Mathematically, the dose for a structure computed from the optimal plan is expressed as in the following:

$$A_{i}x=d_{i}$$ (1)

where *A*_*i*_ is the system matrix of structure *i*, *x* (source dwelling time) is the optimal plan to be optimized, and *d*_*i*_ is the dose distribution for structure *i*. The system matrix is usually called the dose kernel matrix in treatment planning. Generating the dose kernel matrix is essential for inverse planning, which was created from the MC-calculated dose distribution in this study.

**Fig 2:**
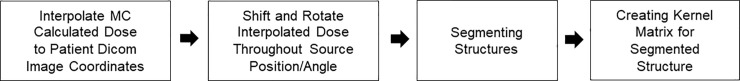
Creation of dose kernel matrix. Procedure to create dose kernel matrix from MC-simulated dose distribution for inverse planning.

**Fig 3:**
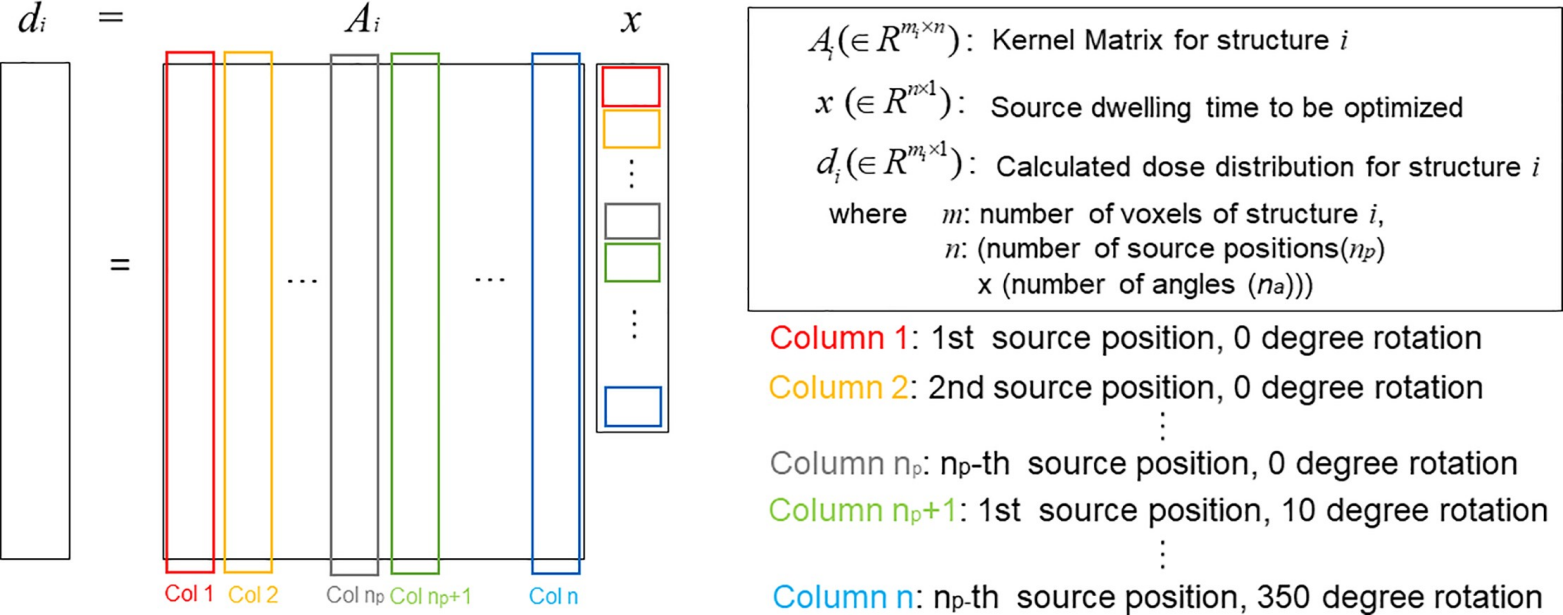
Formation of dose kernel matrix for each structure. Dose kernel matrix for each structure, where the dose distribution for each structure fills column of the matrix in a 1D-array.

The procedure to create the dose kernel matrix for each structure is summarized in Fig 2. To be specific, the MC-simulated dose needs to be interpolated to the coordinates for the given patient image (MR/CT). The catheter-reconstructed path for the applicator can be slightly oblique to the axial plane, which might be different from the MC-calculated dose that is assumed to be orthogonal to the axial plane. Thus, the dose distribution also needs to be tilted and rotated considering the applicator pathway. The interpolated dose can be translated and rotated according to the pre-determined source position and the degree of rotation. The shifted and rotated dose needs to be segmented by structure delineations produced by oncologists, which finally formulate the structure-wise dose kernel matrix by stretching the elements to a one-dimensional array as described in Fig 3.

### Plan optimization by L0-norm regularization

The novel tandem applicator in Fig 1 has a single channel, which requires more traveling frequencies as the source dwell positions and angles for intensity modulation increase. This is undesirable with respect to the delivery efficiency. Of various considerations required for plan optimization, the most important factor for the new applicator is the reduction of the number of optimal source dwell points while preserving the plan quality.

Reducing the number of source dwell points (position and angles) means that there must be few meaningful, non-zero elements in solution *x*. Specifically, this problem is cast as minimizing the number of non-zero elements, which is equivalent to the objective of sparse signal recovery. Thus far, the absolute sum of the elements has mostly been used to solve the sparse signal recovery problem, namely L1-norm minimization, because it is a tractable, convex optimization problem [21–26]. However, the ideal solution to sparse signal reconstruction is minimization of L0-norm whose definition is to count the number of non-zero elements. The objective, thus, could be expressed as the following regularized form:

$$\underset{x}{\min.}\sum_{i=1}^{N}\alpha_{i}\|A_{i}x-d_{i}{\|}_{2}^{2}+\lambda \|x{\|}_{0}$$ (2)

where *α*_*i*_ represents the important factors for different organs, *x* (*x*∈*R*^*n*×1^) is the source dwelling time at *n* pre-determined source dwell points, *A*_*i*_ is the dose kernel matrix ($A_{i}\in R^{m_{i}\times n}$, *m*_*i*_ is the number of voxels of structure *i*), *d*_*i*_ is the desired dose distribution of structure *i* ($d_{i}\in R^{m_{i}\times 1}$), and λ represents the regularizing parameter. The first term is intended to minimize the differences between the ideal and computed dose distribution, while the second term competes with the first term to reduce the number of meaningful source dwell points in dose optimization.

In practice, however, converging the problem with L0-norm minimization is difficult due to the non-convex nature. A couple of techniques are available to make the problem solvable by using alternating direction algorithms and primal-dual solvers [23, 25]. This work attempted to solve the problem by modifying one of the primal-dual solvers called the Chambolle-Pock algorithm [26]. Assuming N = 1 and *α*_*i*_ = 1, the mathematical model can be written in the following form:

$$\begin{array}{l} F(Kx)+G(x),\;\mathrm{where}\;K=\left[\begin{array}{c} A_{i}\\ \lambda I \end{array}\right],\;G(x)={\left[x\right]}_{+}\\ \;F(Kx)=\left[\begin{array}{l} f_{1}(A_{i}x)\\ f_{2}(x) \end{array}\right]=\left[\begin{array}{l} \|A_{i}x-d_{i}{\|}_{2}^{2}\\ \lambda \|x{\|}_{0} \end{array}\right] \end{array}$$ (3)

where [*x*]_+_ is the function that takes the positive value only in *x*. To handle the possibly non-convex function, a modified Chambolle-Pock algorithm was proposed by [27], which updated the variables as follows:

$$y_{l}{}^{n+1}=prox_{\sigma^{-1}F}(\sigma^{-1}y_{l}{}^{n}+Kx^{n})$$ (4)

$$z_{l}{}^{n+1}=z_{l}{}^{n}+\sigma (Kx^{n}-y_{l}{}^{n+1})$$ (5)

$$x^{n+1}=prox_{\tau G}(x^{n}-\tau \sum_{l}K_{l}{}^{T}z_{l}{}^{n+1})$$ (6)

where *y* and *z* are the dual variables used to update the primal solution *x*, and *σ* and *τ* are the algorithmic coefficients to be assigned. This work applied *σ = 0*.*8*, *τ* = 0.0001 and λ = 50 for implementation. Algorithm I summarize the algorithmic flow and variable updates implemented in this work.

### Plan optimization by group sparsity

The L0-norm regularization in the preceding section could produce very few dwell points, thus leading to more efficient delivery. However, this might lack the consideration of how the new applicator works with the afterloader synchronized. The treatment is held until the applicator is placed at a scheduled rotation angle. Once the servo motor rotates the applicator to the scheduled angle, the system clears the interlock by pushing the ‘start’ button. Then, the afterloader puts the radiation source into the designated positions, and pulls it out after the planned dwell time. The same procedure repeats on the subsequent rotational angles. That is analogous to the static fixed-beam IMRT in operating principle. Thus, to enhance the delivery efficiency effectively, the number of rotational angles needs to be minimized.

The issue of making the number of rotational angles sparse has been studied in the beam orientation optimization (BOO) in external beam radiotherapy [28, 29, 33, 34]. The recent developments[28, 29, 34] adopted an algorithm, called a group sparsity, that minimizes the number of beam angles in external beam radiation therapy. This work attempts to add the group sparsity constraint to the L0-norm regularization as expressed in the following:

$$\begin{array}{l} \underset{x}{\min.}f(x)=\sum_{i=1}^{N}\alpha_{i}\|A_{i}x-d_{i}{\|}_{2}^{2}+\lambda \|x{\|}_{0}+\sum_{b=1}^{B}w_{b}\|x_{b}{\|}_{2}^{p}+I_{\geq 0}(x)\\ \mathrm{where}\;I_{\geq 0}(x)=\{\begin{array}{cc} 0 & \mathrm{if}\;x\geq 0\\ \infty & \mathrm{otherwise} \end{array} \end{array}$$ (7)

where $\sum_{b=1}^{B}w_{b}\|x_{b}{\|}_{2}^{p}$ with L2,*p*-norm (0≤*p*<1) is the group sparsity that plays a role as minimizing the number of necessary rotational beam angles, and *w*_*b*_ is the regularizing parameter for a beam angle *b*. It is defined as:

$$w_{b}=c{\left(\|A_{HR-CTV}^{b}{\|}_{2}\right)}^{q}$$ (8)

where *c* is the constant that controls the active angles, $A_{HR-CTV}^{b}$ is the kernel matrix of the target volume, contributing to the rotational angle *b*, and *q* is the power of the given equation. We set *c* and *q* to be 200 and 0.25 in this work, where the value of *q* does not affect the result substantially once it is less than 2.

The Eq (7) to be solved is a combination of differentiable and non-differentiable terms, even comprehending possibly non-convex group sparsity term if *p* is less than 1. This type of problem is usually solved by a proximal gradient method, recently combining the accelerations [24]. To do so, it is decomposed into two functions as follows:

$$\begin{array}{l} \underset{x}{\mathrm{minimize}}\;f(x)=g(x)+h(x),\\ \;\left\{ \begin{array}{l} g(x)=\sum_{i=1}^{N}\alpha_{i}\|A_{i}x-d_{i}{\|}_{2}^{2}\\ h(x)=\lambda \|x{\|}_{0}+\sum_{b=1}^{B}w_{b}\|x_{b}{\|}_{2}^{p}+I_{\geq 0}(x) \end{array}\right. \end{array}$$ (9)

where *g*(*x*) is the differentiable function, and *h*(*x*) is the function that contains non-differentiable, non-convex terms. And *B* is the maximum number of rotational angles for the applicator, and *x*_*b*_ is the vector of source dwell time associated with the rotational angle *b* (*b∈B*). As *h*(*x*) includes two convex terms, it should be difficult to be solved. Thus, the L0-norm was replaced with the reweighted L1-norm, which is an approximated algorithm combined with the tractable L1-norm [35]. It could be expressed in the following:

$$\begin{array}{l} h(x)=\lambda \|Rx{\|}_{1}+\sum_{b=1}^{B}w_{b}\|x_{b}{\|}_{2}^{p}+I_{\geq 0}(x)\\ \left(r_{i}=\frac{1}{|x_{i}|+\delta },\|Rx{\|}_{1}=\sum_{i}\frac{\left|x_{i}\right|}{|x_{i}|+\delta }\approx \sum_{i}1\{x_{i}\neq 0\}\right) \end{array}$$ (10)

*R* is the reweighting matrix that approximates to the L0-norm if the positive coefficient *δ* is sufficiently small, where the elements were defined to be unity, and updated after the initial optimization (*δ* was set to be 10% of the maximum of *x*). It requires for the additional iteration to reach the optimal solution. We reiterated it with only one additional time. Considering the fact that *x* and *R* is the concatenation of the elements in rotational angle *b* (*x*_*b*_, and *R*_*b*_), *h*(*x*) can be expressed as:

$$\begin{array}{l} h(x)=\sum_{b=1}^{B}h(x_{b}),\;\mathrm{where}\\ h(x_{b})=\lambda \|R_{b}x_{b}{\|}_{1}+w_{b}\|x_{b}{\|}_{2}^{p}+I_{\geq 0}(x_{b}) \end{array}$$ (11)

The function *h*(*x*) is regarded as a proximity operator for each iteration. The source dwell time (beam weight) is optimized by the accelerated proximal gradient algorithm as shown in the following:

$$y=x^{k}+\frac{k}{k+3}(x^{k}-x^{k-1})$$ (12)

$$x^{k+1}=prox_{th}(z),\;(z=y-t\nabla g(y))$$ (13)

$$x_{b}{}^{k+1}=prox_{th}(z_{b})=prox_{tw_{b}\|\cdot {\|}_{2}^{p}}(\max(z_{b}-\lambda t\cdot R_{b},0))$$ (14)

The algorithmic details about the group sparsity is specified in Algorithm II and the previous publications [28, 29]. The performance of the group sparsity in L2,*p*-norm (0≤*p*<1) depends on how *p* is determined. We tried to test the algorithm with both *p* = 0 and *p* = 1/2. This work chose *p* to be 1/2 as the hyper-parameters were more consistent depending on the datasets with no significant degeneracy in algorithmic performance.

### Evaluation

The proposed algorithms and inverse planning framework were validated using retrospective data for six pre-treated patients. As this is a retrospective study, institutional review board (IRB) waived the need for informed patient consent to the use of patient data including image, structure, dose and planning information, which was approved by the National Cancer Center, Korea (NCC2020-052). The cervical cancer patients who had asymmetric shape of the clinical target volume relative to cervical canal were selected, such that the effect of intensity modulation could be investigated. All patient data were acquired from treatment planning system, and anonymized before analysis. Among them, five cases were MR-guided and one involved CT-based brachytherapy. The MR and CT images used for this study had 512x512x40 voxels with 0.51x0.51x4.0 mm^3^ resolution and 512x512x65 voxels with 0.51x0.51x4.0 mm^3^ resolution, respectively. The radiation sources were assumed to be placed with a distance interval of 2.5 mm and an angular distance of 10^o^ on the reconstructed catheter path, which led to total 36 candidates for rotational angles. Source alignment resulted in approximately 600 pre-determined source positions and angles depending on the patient HR-CTV anatomy. The structures included in the optimization were HR-CTV as the target, and the bladder, rectum, bowel, and sigmoid as critical organs-at-risk.

For each case, plans from the rotatable tandem applicator were compared against conventional intracavitary brachytherapy plans. The plans were optimized without the ovoid applicator, which might lead to clinically acceptable plan quality in some cases. But this was designed to clarify the effect from intensity modulation by the new, proposed tandem applicator. Additionally, the algorithmic performance of the L0-norm-constrained in (2) and group-sparsity algorithms in (7) was evaluated in terms of delivery efficiency and plan quality relative to plan optimized from the unconstrained algorithm in (15):

$$Unconstrained:\;\underset{x}{\min.}\;\sum_{i=1}^{N}\alpha_{i}\|A_{i}x-d_{i}{\|}_{2}^{2}$$ (15)

All optimized plans were normalized such that 90% of the HR-CTV received the prescribed radiation dose. As the proposed algorithms based on the L0-norm only and group sparsity with L0-norm constraints intended to enhance delivery efficiency, the algorithmic performance was demonstrated by the entire number of source loading points, and the number of active rotational angles for the applicator. Under the design of the control system, fewer rotational angles should be likely to enhance actual delivery efficiency. The plan quality was assessed using dose volume histograms (DVHs), the dose at 2 cc for each structure, and the dose distribution.

Our implemented algorithms were executed on a PC with 16 GB DDR3 memory and 2.5 GHz Intel Core i7 processor. The number of iterations allowed was 10,000 times, and the calculations stopped if $\|x^{n+1}-x^{n}{\|}_{2}/\|x^{n+1}{\|}_{2}\leq 10^{-4}$. With this computational unit, the time elapsed for the plan optimization was about 5 minutes for the L0-norm constrained and 2 minutes for the group sparsity with L0-norm constrained algorithm, respectively, while varying the time depending on the voxel sizes of the target and normal tissue volumes. It could be made much more efficient by employing the graphics processing unit (GPU). The hyper parameters used for Algorithm I was empirically determined to ensure the optimal planning result. *σ* and *τ* were 10^−4^ and 0.6, respectively, and *λ* ranged from 20 to 50. The parameters *t* and λ for Algorithm II were defined to be 1.5e-4, and 20 to 50, respectively.

**Algorithm I.** Modified primal-dual method for L0-norm regularization

    Initialize *x*^0^≔0, *σ*^1^⋅*τ*^1^⋅‖K‖^2^≤1

    for *k* = 1,2, …

        1. Update *y*:

            $y_{1}{}^{n+1}=prox_{\sigma^{-1}F}(\sigma^{-1}z_{1}{}^{n}+A_{i}x^{n})=\frac{A_{i}x^{n}+\sigma^{-1}(z_{1}{}^{n}+d_{i})}{1+\sigma^{-1}}$

            $y_{2}{}^{n+1}=prox_{\sigma^{-1}F}(\sigma^{-1}z_{2}{}^{n}+\lambda x^{n})=prox_{\sigma^{-1}F}({\hat{y}}_{2}{}^{n})=\left\{ \begin{array}{l} {\hat{y}}_{2}{}^{n}\;(|{\hat{y}}_{2}{}^{n}{|}_{i}\geq \sqrt{2\lambda \sigma^{-1}})\\ 0\;(|{\hat{y}}_{2}{}^{n}{|}_{i}<\sqrt{2\lambda \sigma^{-1}}) \end{array}\right.$

        2. Update *z*:

            $z_{1}{}^{n+1}=z_{1}{}^{n}+\sigma (A_{i}x^{n}-y_{1}{}^{n+1}),z_{2}{}^{n+1}=z_{2}{}^{n}+\sigma (\lambda x^{n}-y_{2}{}^{n+1})$

        3. Update *x*:

            $x^{n+1}=prox_{\tau G}(x^{n}-\tau \sum_{l}K_{l}{}^{T}z_{l}{}^{n+1})={\left[x^{n}-\tau (A_{i}{}^{T}z_{1}{}^{n+1}+\lambda z_{2}{}^{n+1})\right]}_{+}$

    end

**Algorithm II.** Accelerated proximal gradient method for group sparsity with L2,*p*-norm

Initialize *x*^0^≔0,*R*≔1,*t*,*λ*,*w*_*b*_

    for *iter* = 1:*max*. *iterations*

        $\mathrm{if}\;iter>1:r_{i}=\frac{1}{|x_{i}|+\delta }$ (Update the element of matrix *R*)

        for *k* = 1,2, …

            $y=x^{k}+\frac{k}{k+3}(x^{k}-x^{k-1})$

            *x*^*k*+1^ = *prox*_*th*_(*y*−*t*∇*g*(*y*))

                *z*←*y*−*t*∇*g*(*y*) (*z*_*b*_:elements in applicator at rotation angle *b*)

                    $x_{b}{}^{k+1}=prox_{th}(z_{b})=prox_{tw_{b}\|\cdot {\|}_{2}^{p}}(\max(z_{b}-\lambda t\cdot R_{b},0))$

                where $p=0:prox_{tw_{b}\|\cdot {\|}_{2}^{0}}(u)=\{\begin{array}{cc} u, & \mathrm{if}\|u{\|}_{2}\geq \sqrt{2tw_{b}}\\ 0, & \mathrm{otherwise} \end{array}$

                        $\begin{array}{l} p=\frac{1}{2}:prox_{tw_{b}\|\cdot {\|}_{2}^{\frac{1}{2}}}(u)\\ \;=\{\begin{array}{cc} 0, & \mathrm{if}\;\|u{\|}_{2}^{-1.5}>\frac{2\sqrt{6}}{9}\\ u{\left(\frac{2}{\sqrt{3}}\sin\left(\frac{1}{3}\left(\arccos\left(\frac{3\sqrt{3}}{4}tw_{b}\|u{\|}_{2}^{-1.5}\right)+\frac{\pi }{2}\right)\right)\right)}^{2}, & \mathrm{otherwise} \end{array} \end{array}$

        end

    end

## Results

**Fig 4:**
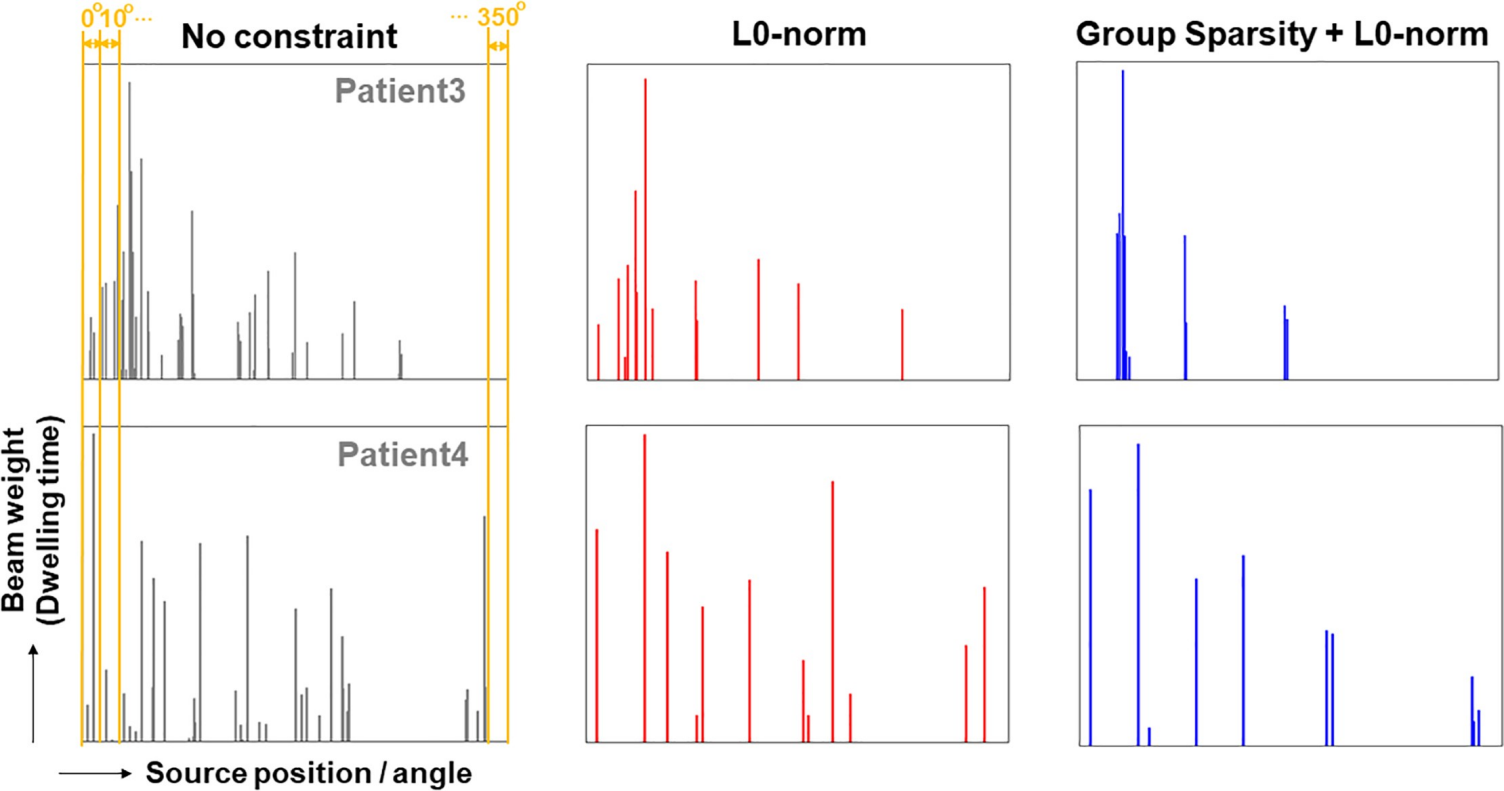
Optimized dwell time and dwell points from three different algorithms. Resulting plans from three different algorithms (unconstrained, L0- and group sparsity constrained) for patients 3 and 4.). The order of x-axis represents the applicator angles and locations. At each applicator rotation angle (0^o^, 10^o^, 20 ^o^, and so on), the bar in the y-axis means the dwell time (intensity) for the designated source positions located in 2.5mm interval.

Fig 4 shows the resulting plans with three different optimizing algorithms for two patient cases (patients 3 and 4), where the order in x-axis in Fig 4 represents the source dwell points under the applicator angles and the bar in y-axis means the source dwell time (weight) for the designated points. It demonstrates that the algorithms of the L0-norm regularization and the group sparsity with L2,1/2-norm yielded the plans with much fewer source loading points. From the 6 cases tested in this work, the L0-norm and group-sparsity constrained algorithms successfully lowered the source dwell points by 61% and 68% relative to the unconstrained algorithm, on average, as seen in Table 1. When comparing the L0-norm and group sparsity constrained algorithms, the group sparsity with L2,1/2-norm reduced the number of entire source dwell points optimized further than the L0-norm constrained.

**Table 1:** Algorithmic performance in efficiency.

	Number of source dwell points			Number of active rotational angles		
	^a^Unconst.	L0-norm	^b^GS+L0	Unconst.	L0-norm	GS+L0
**Patient1**	25	10	9	6	4	4
**Patient2**	22	13	11	7	5	4
**Patient3**	47	13	10	17	9	4
**Patient4**	38	12	10	18	10	6
**Patient5**	35	14	12	16	9	5
**Patient6**	16	9	7	12	8	5
**Avg.**	30.5	11.8	9.8	12.7	7.5	4.7
**Avg. (patient 3–6)**	34.0	12.0	9.8	15.8	9.0	5.0

Comparing performance of three different optimization algorithms in number of source dwell points, and number of active rotational angles that possibly reflect the delivery efficiency

^a^Unconst: Unconstrained optimization

^b^GS+L0: (Group sparsity + L0-norm) constrained algorithm

As stated in the previous section, the currently developing control system of the rotational IMBT behaves similar to the static IMRT. Due to the structural features, the number of active rotational angles should affect the delivery efficiency more directly than the entire number of source dwell points. Table 1 also lists up the number of active rotational angles of the three plans optimized from different algorithms. The first two cases show that the reduced number of source dwell points does not guarantee the decrease in the active rotational angles. Although the proposed algorithms substantially reduced the source dwell points, the number of active rotational angles remains similar. For the remaining four cases (excluding the first two cases), however, our proposed algorithms reduced the number of active rotational angles by 43%, and 68%, respectively, which amount to almost saving 7 to 11 applicator rotating angles, relative to the unconstrained algorithms.

**Fig 5:**
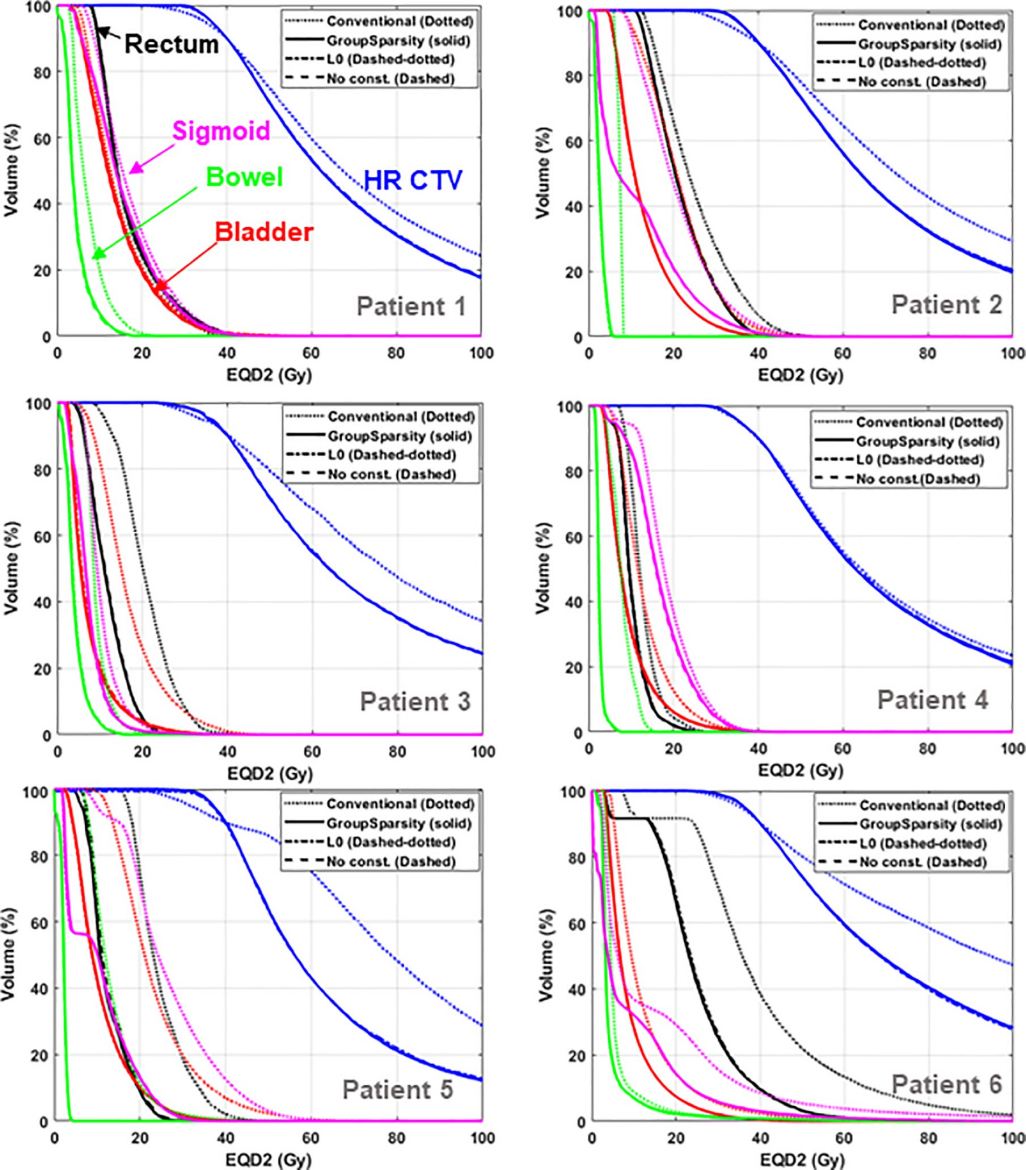
Dose volume histograms of optimized plans. Comparison of DVHs from conventional (isotropic) tandem applicator-based brachytherapy plan (dotted), and new rotational tandem-based brachytherapy plans optimized using L0-norm-constrained (solid), L1-norm-constrained (dashed-dotted), and unconstrained (dashed) algorithms for six test cases.

Besides enhancing the delivery efficiency by decreasing the source loading points, it is important to see if the plan with a smaller number of source dwell points and/or fewer active rotational angles can preserve the plan quality. Thus, the resulting plans with different numbers of source dwell points from different algorithms (referring to Table 1) were evaluated using DVHs as illustrated in Fig 5, including all planning results from the three different algorithms. For a fair comparison, the plans were normalized to cover the HR CTV by 90% with the prescribed dose, which was 40 Gy in this work. The three algorithms appeared to produce similar results in the DVHs of HR-CTV and other critical organs such as bladder, rectum, bowel, and sigmoid for the 6 test cases. Importantly, the three plans optimized with the new, rotatable tandem applicator were also compared against those from the conventional tandem-based intracavitary brachytherapy plans. The results showed that the intensity modulation significantly enhanced the plan quality for all test cases.

**Fig 6:**
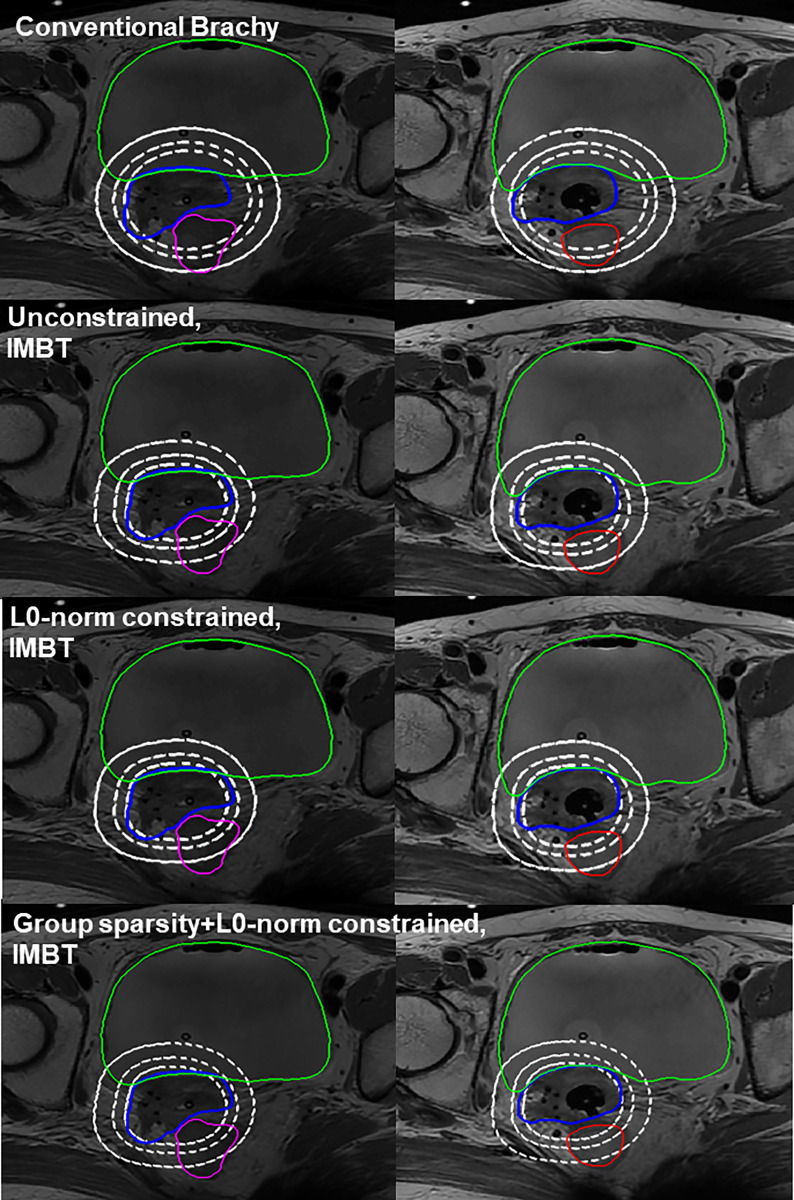
Dose distributions of optimized plans. Comparison of dose distributions of patient 6 in two axial slices from conventional brachytherapy plan, and three IMBT plans from unconstrained, L0-norm and (group sparsity+L0-norm) constrained algorithms with 16, 9, and 7 dwell points, and 11, 8 and 5 active angles optimized, respectively, where three iso-dose lines correspond to 50, 75, and 100% of the prescribed dose (HR-CTV, bladder, sigmoid, and rectum in blue, green, magenta, and red, respectively).

We also visualized the dose distributions on two axial slices for patient 6 and compared four different plans: the conventional tandem-based plan and three new rotational tandem-based plans with different numbers of source loading points (16, 9 and 7 dwell points) and active rotational angles (12, 8 and 5 angles) obtained from three algorithms. The effects of intensity modulation are shown in Fig 6. The dose distributions from the new tandem applicator on the bottom three rows better conform to the target volume (HR-CTV in blue) and spare the critical organs (bladder in green, sigmoid in magenta, and rectum in red) relative to the dose distribution on the top row from the conventional brachytherapy plan. Of note, the dose distributions on the right three images are with similar doses, implying that the proposed algorithm has sufficiently great algorithmic performance even with fewer dwell points applied.

Tables 2 and 3 list the detailed, numerical information about the quality of the conventional intracavitary brachytherapy and three optimized plans with the new tandem applicator by measuring the dose irradiated to 2 cc (D_2cc_) for each structure, and the volume of HR-CTV irradiated by 100, 150, and 200% of the prescribed dose. The values shown in Tables 2 and 3 represent the percent dose relative to the target prescribed dose and volume in cc, respectively. In most cases, the dose of radiation in the proposed plan deviates by less than 2% from the plan optimized by the unconstrained and L1-constrained algorithms. As seen in Table 2, the three IMBT plans from the different algorithms had similar volume (cc) irradiated by the designated dose. Contrarily, the brachytherapy plan with conventional tandem resulted in higher dose irradiated to 2 cc and greater volume exposed to the same amount of radiation. The Student’s paired T-test or Wilcoxon signed rank test was applied to each structure to verify if the difference in the plan quality produced from the group sparsity constrained against the others is statistically significant. For all structures evaluated, the differences between the IMBT plans from L0-norm and the conventional brachytherapy plans were found statistically significant. In contrast, for the IMBT plans from three different algorithms, the planning results were demonstrated to be statistically similar according to the p-value seen at the bottom of Table 2. The same trend was shown in Table 3 when analyzing the volume of HR-CTV irradiated by 100, 150, and 200% of the prescribed dose.

**Table 2:** D_2cc_ of the optimized plans.

Data	Algorithm	HR-CTV	Bladder	Rectum	Bowel	Sigmoid
Patient 1	^a^Conv. Intra.	296.00	40.89	31.95	17.78	36.44
	No const.	254.91	39.35	31.88	12.56	33.69
	L0-const.	264.63	40.60	31.48	12.80	33.98
	GS+L0-const.	253.75	40.09	31.65	12.91	33.64
Patient 2	Conv. Intra.	341.17	47.06	97.65	9.41	45.88
	No const.	299.58	37.12	36.33	5.18	38.43
	L0-const.	303.41	36.76	35.53	5.26	39.26
	GS+L0-const.	295.16	37.02	35.86	5.21	38.73
Patient 3	Conv. Intra.	262.22	42.66	32.89	17.78	22.22
	No const.	225.66	30.42	19.84	11.32	16.50
	L0-const.	235.62	29.32	20.02	11.32	16.64
	GS+L0-const.	228.16	29.56	19.98	11.29	16.81
Patient 4	Conv. Intra.	205.00	36.43	19.29	15.00	33.57
	No const.	198.87	33.94	15.30	6.08	31.66
	L0-const.	197.68	33.85	15.68	5.76	31.32
	GS+L0-const.	200.75	33.84	15.85	5.94	31.85
Patient 5	Conv. Intra.	145.14	59.43	32.00	30.86	51.43
	No const.	92.59	36.66	18.01	3.42	28.22
	L0-const.	95.24	36.05	17.64	3.52	28.09
	GS+L0-const.	92.21	36.53	17.63	3.51	28.00
Patient 6	Conv. Intra.	364.55	55.45	63.64	22.73	63.64
	No const.	237.78	38.41	37.99	18.70	37.46
	L0-const.	235.67	38.15	38.20	18.43	37.09
	GS+L0-const.	240.85	38.33	38.09	18.97	37.72
T-test (p-value)	GS+L0-const vs. Conv. Intra.	0.031	0.031	0.031	0.031	0.031
	GS+L0-vs. No const.	0.844	0.313	0.844	0.344	0.219
	GS+L0- vs. L0-const	0.313	0.563	0.406	0.438	0.719

D_2cc_ of conventional intracavitary brachytherapy plan and three IMBT plans from different algorithms for HR-CTV, Bladder, Rectum, Bowel, and Sigmoid (% dose relative to prescribed dose)

^a^Conv. Intra.: Conventional intracavitary brachytherapy plan

**Table 3:** V_100%_, V_150%_, and V_200%_ of the optimized plans.

Data	Algorithm	HR-CTV		
		^a^V_100%_	^b^V_150%_	^c^V_200%_
Patient 1	^a^Conv. Intra.	79.18	53.15	32.81
	No const.	79.39	46.61	26.96
	L0-const.	79.34	47.23	27.20
	GS+L0-const.	79.35	46.61	26.77
Patient 2	Conv. Intra.	81.99	58.95	39.01
	No const.	82.10	51.08	29.43
	L0-const.	82.10	51.30	29.76
	GS+L0-const.	82.16	51.27	29.36
Patient 3	Conv. Intra.	33.76	25.54	17.64
	No const.	33.59	20.55	12.98
	L0-const.	33.60	20.68	13.11
	GS+L0-const.	33.55	20.39	13.19
Patient 4	Conv. Intra.	36.57	22.61	14.17
	No const.	36.78	22.18	13.56
	L0-const.	36.75	22.21	13.58
	GS+L0-const.	36.79	21.98	13.32
Patient 5	Conv. Intra.	16.68	13.81	8.95
	No const.	16.67	7.98	3.93
	L0-const.	16.60	7.98	4.07
	GS+L0-const.	16.70	7.97	3.92
Patient 6	Conv. Intra.	34.97	27.78	22.65
	No const.	34.9	22.93	15.50
	L0-const.	34.89	22.94	15.41
	GS+L0-const.	34.87	23.04	15.72
T-test (p-value)	GS+L0-const vs. Conv. Intra.	0.563	0.031	0.031
	GS+L0-vs. No const.	0.438	0.813	0.422
	GS+L0- vs. L0-const	0.453	0.156	0.156

V_100%_, V_150%_, and V_200%_ in HR-CTV of conventional intracavitary brachytherapy and three IMBT plans from different algorithms (cc)

^a^V_100%,_

^b^V_150%,_

^c^V_200%_: Volume of the structure that receive 100%, 150%, 200% of the prescribed dose

## Discussion

The new tandem for IMBT is characterized by beam intensity modulation with a single channel through applicator rotation with 0.1^o^ precision. Dosimetric benefits from the proposed rotational IMBT device over the conventional tandem-based brachytherapy were investigated and verified in our previous work. Considering the structural features of the rotational IMBT tandem, a couple of aspects need to be resolved before the actual treatment: 1) requirement of inverse planning and 2) optimization with minimal source dwelling angles/positions on the inverse planning framework.

This work focused on finding a plan with minimum number of source dwell points and angles from inverse planning as it is important not only for efficient dose delivery but also for patient safety that can reduce the source transit time, thus decreasing unnecessary dose to the patient. The inverse planning process was developed using an MC-simulated dose distribution on a specific source loading position orthogonal to the axial plane with no rotation. The 3D MC-simulated dose distribution was translated and rotated by the tandem geometry and structure delineation was performed, which led to creating a kernel matrix for the structures, including the target volume and critical organs. With the resulting kernel matrix, we implemented two optimization algorithms that can ensure a minimal number of source loading points (position/angle).

Finding a solution with the fewest dwell points is mathematical equivalent to making the solution have the fewest non-zero elements possible (as sparse as possible). This notion corresponds to the definition of L0-norm, which is an ideal solver for sparse signal reconstruction. Despite the challenges from its non-convexity, the recent algorithmic, theoretical progress enables solving the problem by a modified Chambolle-Pock primal-dual method. The L0-norm regularization only, however, may yield sub-optimal results in delivery efficiency as it did not fully reflect the operating principle and physical constraints of the new IMBT applicator that is similar to the static fixed-beam IMRT. Hence, this work added the group sparsity in L2,*p*-norm (0≤*p*<1) to the L0-norm constraint to reduce the number of active applicator rotating angles. The previous studies implemented the group sparsity algorithm with the L1-norm constraint to promote the element sparsity, which resulted in many source dwell points although it lowered the active rotating angles. Though not comprehended in the results, the L1-norm constrained algorithm produced 35% more dwell points and 3.5 more active angles on average than the L0-norm constrained only. Thus, we combined the group sparsity with the L0-norm constraint which makes the problem hard to be solved as there exist two non-convex terms inside. The problem was addressed by an approximation to L0-norm from the reweighting matrix with traceable L1-norm at the cost of additional iterates.

From the six retrospective patient data with 2.5 mm interval and 10^o^ angular distance between consecutive control points, it was demonstrated that the proposed algorithms followed by the inverse planning framework successfully decreased the number of meaningful source dwell points, and active rotational angles. Relative to the unconstrained algorithm, the L0-norm and group sparsity constrained algorithms reduced the number of non-zero elements by about 60% and 70%, and the number of active rotational angles by about 40% and 60% on average, respectively. The degree of enhancement in the number of both source dwell points and angles was greater on the group sparsity constrained with L0-norm regularization than the L0-norm constrained only. Mathematically, the bigger enhancement is interpreted that an angular dependent coefficient, *w*_*b*_ in (7), in group sparsity contributes to eliminating relatively unimportant rotating angles. The results above, therefore, make sense to support our hypothesis that the group sparsity constraint would be more effective in improving the delivery efficiency as it involves the physical constraint appropriately. Of note, the plans with much fewer dwell positions and angles did not compromise the plan quality, compared against the unconstrained algorithm. Also, this study demonstrated the dosimetric benefits of the novel rotational tandem applicator mainly from intensity modulation by comparing the optimized IMBT plans against the conventional tandem-based brachytherapy plans.

In the current phase of development, the new apparatus equipped with the servomotor rotating the radiation shield inside the tandem applicator has been made to be integrated into the control system. It aims to synchronize the applicator to the afterloader, such that the dose is delivered throughout the afterloader when the servo motor rotates the applicator to the planned angles and interlock is held off. Unfortunately, due to the ongoing development, this did not provide entire treatment time for patient specific fashion. For reference, the actual dose delivery time from tandem for 5 Gy HDR plan with Ir-192 source (7.5Ci) is about 5 to 10 minutes, depending on the target size. Eliminating the unnecessary source dwell points by employing the mathematical constraints slightly decreases the degree of beam modulation, therefore reducing the entire source dwell time by 1% or less on average, relative to the unconstrained. As discussed earlier, the primary part of improving the delivery efficiency stems from the decrease in the number of active rotational angles, therefore reducing the source travel time by the afterloader, and the interlocks required for applicator rotation by servo motor and user (therapist) operations. From our observations, the L0-norm and group sparsity constrained algorithms led to almost 7–10 fewer angles than the unconstrained for the highly modulated cases (excluding the first two patients with very few active rotational angles), which may amount to saving a couple of minutes expectedly. The effort to reduce the source dwell positions and angles should be promoted not only for delivery efficiency, but also for patient safety by decreasing transit dose to the patients and lower dose uncertainty with more prominent source dwell points.

This work focused on the development of technical aspects such as inverse planning and plan optimization, so that the rotational tandem applicator is guaranteed to yield optimal, efficient treatment plan. For the proposed framework to be clinically applicable, the essential procedure is to verify the accuracy of the dose delivered to the patient, namely patient specific quality assurance (QA). Additionally, a program comprehending extraction of patient DICOM files, creation of kernel matrices, and optimization of source dwell time, position, and angles needs to be established for clinical usage. Hence, the subsequent work pursues an integrating package of the novel tandem applicator, and treatment planning system.

## Conclusions

The purpose of this work is to construct a series of workflows, including inverse planning and plan optimization, tailored to a novel, rotational IMBT tandem applicator. The article first proposes a framework for inverse planning, which creates kernel matrices employed for plan optimization. The ultimate goal of the proposed plan optimization algorithms based on L0-norm and group sparsity constraints, respectively, is to minimize the number of source dwell points and active rotational angles, such that it could enhance the delivery efficiency by eliminating unnecessary source dwell points without compromising the plan quality. Using data for six patients, it was demonstrated that the plan quality was maintained even with 60–70% reduction of the source loading points, and with 5–8 fewer active angles (7–11 fewer angles for the highly modulated cases), relative to the unconstrained optimizations. It also proved that the optimized IMBT plans outperform the conventional tandem applicator-based brachytherapy in plan quality from the 6 cases tested.

## Supporting information

S1 Data (ZIP)

S2 Data (ZIP)
